# Non-Exercise Based Estimation of Cardiorespiratory Fitness Mediates Associations between Comorbidities and Health-Related Quality of Life in Older Korean Adults with Diabetes

**DOI:** 10.3390/ijerph17041164

**Published:** 2020-02-12

**Authors:** Inhwan Lee, Shinuk Kim, Hyunsik Kang

**Affiliations:** 1 College of Sport Science, Sungkyunkwan University, Suwon 16419, Korea; ansh00@skku.edu; 2 College of Kyedang General Education, Sangmyung University, Cheonan 31066, Korea; kshinuk@gmail.com

**Keywords:** physical fitness, quality of life, chronic diseases, Korean adults

## Abstract

This study investigated whether non-exercise-based estimation of cardiorespiratory fitness (eCRF) mediates the association between health-related quality of life (HRQoL) and comorbidities in older Korean adults with diabetes. A total of 1371 Korean adults (56% women) aged 60 years and older with diabetes was drawn from those who participated in the 2008–2011 Korea National Health and Nutrition Examination Surveys IV and V. Data on comorbidities included hypertension, heart disease (acute myocardial infarction or angina), stroke, arthritis, and chronic renal disease. HRQoL was assessed using the EuroQoL group, which consists of a health-status descriptive system and a visual analogue scale. eCRF was determined with sex-specific algorithms. Age, sex, household income, education level, marital status, smoking, alcohol consumption, and regular exercise were additionally measured as covariates. HRQoL found to be inversely associated with number of comorbidities and positively associated with increasing eCRF category (from low to high) in older Korean patients with diabetes. The Sobel mediation test showed a significant indirect effect (Z = −4.632, *p* < 0.001), and the result of a bootstrap procedure corroborated the Sobel test result: a non-zero range in the 95% bias-corrected confidence interval (95% CI −1.104 to −0.453) indicated that eCRF mediates the impact of comorbidities on HRQoL. Overall, the current findings suggest that enhancing CRF can facilitate positive outcomes, including better HRQoL, for patients with diabetes.

## 1. Introduction

Diabetes represents a clustering of heterogeneous risk factors that affects all aspects of quality of life due to the lifetime demands of diabetes care [1]. Health-related quality of life (HRQoL) refers to a multi-dimensional concept of quality of life, which can be affected by illnesses or treatments. Thus, assessment of HRQoL is an important outcome measure to evaluate overall disease management for patients with diabetes, including disease course, early detection of complications, and effectiveness and efficacy of interventions [2]. 

Comorbidity is defined as the presence of chronic conditions existing concurrently with a primary disease [3]. Patients with diabetes often have multiple comorbidities, including hypertension, overweight or obesity, hyperlipidemia, chronic kidney disease, and cardiovascular disease, all of which can impact the patient’s HRQoL [4,5]. Consequently, the management of chronic conditions in patients with diabetes will benefit from a focus on HRQoL.

Cardiorespiratory fitness (CRF), which is defined as the minimum volume of oxygen consumption during a maximal exercise test, has been found to protect against chronic diseases and premature death from those diseases [6,7]. For example, higher CRF is associated with lower risks of morbidity and mortality in younger and older adults [8]. Some studies have examined the association between physical activity and HRQoL in healthy persons, as well as those with chronic diseases, reporting positive relationships for both groups [9,10]. 

Unlike physical activity (PA), less attention has been paid to the relationship between CRF and HRQoL. Although research in this area is limited, the current evidence suggests a positive relationship between CRF and HRQoL in patients with chronic and metabolic diseases [11]. In older persons, however, objectively measuring CRF can be difficult due to the requirements related to its measurement, such as specialized equipment; trained personnel; amount of time, volitional exertion; and mobility. Importantly, studies have found that CRF can be estimated with acceptable accuracy from routinely obtained health indicators [12,13]. We have previously shown that non-exercise-based estimation of cardiorespiratory fitness (eCRF) can serve as a prognostic tool for estimating the risk of mortality from all and specific causes in older Korean adults [14].

Nationwide data in South Korea revealed that a majority of patients with diabetes have one or more comorbidities which can affect HRQoL, including obesity, hypertension, hypercholesterolemia, and others [15]. Health behaviors, including PA and CRF, likely mediate the impact of comorbidities on HRQoL among these patients. To the best of our knowledge, however, no previous studies have examined the mediating effect of CRF on the relationships between comorbidities and HRQoL in patients with diabetes in South Korea. Therefore, we investigated whether eCRF mediates the impact of comorbidities on HRQoL in older Korean adults with type-2 diabetes.

## 2. Materials and Methods 

### 2.1. Study Design and Participants

The cross-sectional data used for this study were drawn from the Korea National Health and Nutrition Examination Surveys (KNHANES) IV and V, which are nationwide surveys that were conducted from 2008 until 2011 in South Korea. A detailed description of the survey sampling method is available elsewhere [16,17]. For the current study, we initially selected a total of 2757 adults aged 60 years and older from those who participated in the 2008–2011 KNHANES IV and V. We then excluded 1386 individuals because no baseline data were available regarding body composition (*n* = 352), resting heart rate (*n* = 799), PA (*n* = 17), and HRQoL (*n* = 218). Consequently, a total of 1371 older adults with diabetes (604 men; 767 women) were included in the final data analyses. The presence of diabetes was determined with a self-reported questionnaire that asked whether the participants had ever received a diagnosis of diabetes from a physician. The institutional review board of human study reviewed and approved the study protocol participants (SKKU 2017-06-009). Informed consent was obtained from all participants in the study.

### 2.2. Study Variables

#### 2.2.1. Assessment of HRQoL (Dependent Variable, Y) 

HRQoL was assessed with the EuroQoL group, which consists of a health-status descriptive system (EQ-5D) and a visual analogue scale (EQ-VAS). The EQ-5D records the level of self-reported problems in five dimensions: mobility, self-care, usual activities, pain/discomfort, and anxiety/depression [18,19]. Each of the dimensions is assessed based on a single question with three response levels (no problems, some problems, and extreme problems). Scores on the EQ-5D index range from −0.171 to 1, where 1 indicates no problems in any of the five dimensions, zero indicates death, and negative values indicate a health status worse than death. Next, patients report their health status with the EQ-VAS, which involves a VAS ranging from 0 (worst imaginable health) to 100 (best imaginable health) [18]. 

#### 2.2.2. Assessment of Comorbidities (Independent Variable, X)

Participants were asked if they had ever been diagnosed by a physician with any of the following medical condition(s): malignancy, hypertension, heart disease (acute myocardial infarction or angina), stroke, arthritis, and/or chronic renal disease.

#### 2.2.3. Estimation of Cardiorespiratory Fitness (Mediator, M)

Non-exercise-based eCRF was calculated as one-minute peak volume of oxygen consumption (VO_2peak_) in units of metabolic equivalents (METs), in accordance with previously reported procedures [13]:

eCRF (METs) = 2.77 (sex) − 0.10 (age) − 0.17 (BMI) − 0.03 (resting heart rate) + 1.00 (physical activity score) + 18.07.

Once the algorithms were implemented, participants were classified into low (lowest 25%), middle (middle 50%), and high (highest 25%) categories on the basis of sex-specific tertiles of the estimated peak VO_2_ distributions.

#### 2.2.4. Covariates

Measured covariates included age, sex, household income, education level (lower than elementary school, middle/high school, or college or higher), marital status (yes or no), current smoker (never or past/current), frequency of alcohol consumption (more or less than twice per week), and regular exercise (yes or no).

### 2.3. Statistical Analyses

All variables were checked for normality, both visually and through the Kolmogorov–Smirnov test, and subjected to an appropriate transformation, if necessary, prior to statistical analyses. Descriptive statistics are presented as means and standard deviations for continuous variables and as frequencies and percentages for categorical variables. Analysis of variance (ANOVA) was used to test linear trends in outcome variables according to number of comorbidities and eCRF categories.

**Figure 1:**
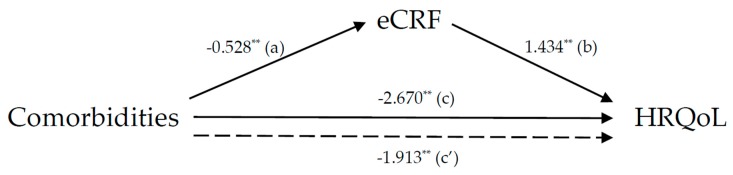
Mediation analysis. Path coefficients of comorbidities on health-related quality of life (HRQoL) through non-exercise based estimation of cardiorespiratory fitness (eCRF). ** indicates statistical significance at *p* < 0.001 for all the paths.

We examined the relationships between number of comorbidities, eCRF, and HRQoL using parametric and non-parametric statistics. Then, the impact of comorbidities on HRQoL through eCRF was tested based on four criteria for the mediation paths proposed by Baron and Kenny [20], as illustrated in Figure 1: (1) the coefficient of path “a” is significant in identifying the effect of the independent variable (IV) on the mediating variable (MV); (2) the MV is significantly related to the dependent variable (DV) of the IV (path b); (3) a significant direct association (path c) between the IV and DV is confirmed; and (4) the association between the IV and DV is weakened when the MV is controlled (path c′). The PROCESS macro in SPSS-PC (version 23.0, IBM Corporation, Armonk, NY, USA) was used to carry out the mediation analyses. No covariates were included in Model 1, demographics and socio-economic status (SES) variables were included in Model 2, and variables of health behaviors were added to Model 3. The possible mediating effect of eCRF on the impact of comorbidities on HRQoL was identified using the Sobel mediation test with a bootstrapping process, which overcomes the assumption of a normal distribution of the classical Sobel mediation test. The robustness of *p*-values was confirmed via the bootstrapping process. An α level of 0.05 was used for all statistical analyses.

## 3. Results 

Table 1 summarizes descriptive statistics of participants. Overall, male participants were older (*p* < 0.001), heavier (*p* < 0.001), more educated (*p* < 0.001), and less likely to be alone (*p* < 0.001) than female participants. Men had higher values for smoking (*p* < 0.001) and alcohol intake (*p* < 0.001), but lower values for resting heart rate (*p* = 0.023) and number of comorbidities (*p* < 0.001) than women. Men were less physically active (*p* < 0.001) but had higher values on the EQ-5D index (*p* < 0.001) and EQ-VAS (*p* < 0.001) than women. We found no significant differences in marital status or income between older men and women. 

Table 2 compares the measured parameters in relation to number of comorbidities. Significantly, positive, linear trends in mean age (*p* < 0.001), solitary status (*p* = 0.022), and smoking status (*p* < 0.001) and significant, negative linear trends in education (*p* = 0.001), alcohol consumption (*p* = 0.004), EQ-5D index (*p* < 0.001), and EQ-VAS score (*p* = 0.002) were found in relation to the number of comorbidities. In general, patients with one or more comorbidities were older, more likely to live alone, less likely to smoke, consumed alcohol less frequently, and had worse HRQoL than patients with no comorbidities. 

Table 3 compares the measured parameters according to eCRF category. Significantly, negative linear trends in mean age (*p* < 0.001), solitary status (*p* = 0.003), physical activity (*p* < 0.001), and number of comorbidities (*p* = 0.008) and significant, positive linear trends in education (*p* = 0.002), alcohol consumption (*p* = 0.034), EQ-5D index (*p* < 0.001), and EQ-VAS score (*p* < 0.001) were found in relation to increased eCRF (from low to high). Patients who were physically fit were younger, less likely to live alone, less physically active, had more education, consumed alcohol more frequently, had fewer comorbidities, and had higher values on the EQ-5D index and EQ-VAS than patients who were less physically fit. No significant linear trends in sex, marital status, income, or smoking were found in relation to eCRF category. 

Table 4 represents the impact of eCRF on the associations between comorbidities and HRQoL. Mediation analysis showed that number of comorbidities had a direct effect on HRQoL independent of eCRF (*β_c’_* = −1.913, *p* < 0.005; *c’* path). However, the number of comorbidities also indirectly affected HRQoL through its effect on eCRF. The number of comorbidities was negatively associated with eCRF (*βa* = −0.528, *p* < 0.001; *a* path), and eCRF was positively associated with HRQoL (*β_b_* = 1.434, *p* < 0.001; *b* path). Both comorbidity and eCRF remained significant predictors of HRQoL even after adjusting for demographics and SES (i.e., age, marital status, living condition, and education in Model 2 (*β_c’_* = −2.327, *p* < 0.001; *c’* path; *β_b_* = 0.996, *p* < 0.001; b path, respectively), as well as health behaviors (i.e., smoking, alcohol consumption, and regular exercise) in Model 3 (*β_c’_* = −2.039, *p* < 0.005; *c’* path and *β_b_* = 1.153, *p* < 0.005; b path, respectively).

The mediating effect of eCRF on the impact of comorbidities on HRQoL was further tested using the Sobel mediation test with a bootstrapping procedure (Table 4 and Figure 1). The Sobel mediation test showed a significant indirect effect of eCRF on the impact of comorbidities on HRQoL (Z = −4.632, *p* < 0.001). The results of the bootstrap procedure corroborated those of the Sobel test: the 95% bias-corrected confidence interval (95% CI −1.104 to −0.453) was non-zero, indicating that eCRF mediated the relationship between comorbidities and HRQoL (Model 1) and accounted for 28.4% of the total effect on HRQoL. The Sobel mediation effect of eCRF on the relationship between comorbidities and HRQoL remained significant even after adjusting for demographics and SES in Model 2 (Z = −3.004, *p* = 0.001), with 16.1% of the total effect being explained. Likewise, this relationship remained significant after adjustments for Model 2 plus inclusion of parameters for health behaviors in Model 3 (Z = −2.753, *p* = 0.005), with 12.1% of the total effect explained.

## 4. Discussion

In this study, we examined the mediating effect of eCRF on the relationships between comorbidities and HRQoL in older Korean adults with diabetes. Our findings show that both comorbidities and eCRF are important predictors of HRQoL among older Korean patients. This is the first study to report that eCRF mediates the impact of comorbidities on HRQoL partially and independently among patients with diabetes.

The current findings agree with previous studies reporting an inverse relationship between comorbidities and HRQoL in patients with diabetes. For example, Wexler et al. [21] examined the impacts of medical comorbidities and depression on HRQoL in a large primary care cohort of patients with type 2 diabetes and showed that treatment of depression and prevention of complications were important determinants of HRQoL. By analyzing the treatment options for type 2 diabetes in adolescents and youth (TODAY) study data, Larkin et al. [22] showed that HRQoL was negatively associated with depressive symptoms and number of comorbidities in youth with type 2 diabetes. This inverse relationship between HRQoL and comorbidities has also been observed in patients with chronic conditions [23], dementia [24], psoriatic arthritis [25], and survivors of breast [26] and colorectal cancer [27].

Likewise, the current findings are consistent with previous studies reporting a positive relationship between CRF and HRQoL in patients with type 2 diabetes [28], pediatric patients with type 1 diabetes [29], and women at risk of gestational diabetes [30]. Using patient records from the 2002 to 2012 Calidad de Vida Center, Clennin et al. [11] examined the relationship between CRF and HRQoL in a Uruguayan cohort at risk for developing cardiovascular disease (CVD) and found positive associations between CRF and several dimensions of HRQoL (women: vitality, physical health, physical role, bodily pain, and general health; men: physical health). In children and adolescents with type 1 diabetes, Lukács et al. [31] examined associations among HRQoL, clinical profiles, anthropometric measures, and physical activity and CRF and found that good CRF was significantly associated with better HRQoL and favorable metabolic control among the youth. Similarly, this positive association between CRF and HRQoL was observed in community-dwelling older adults [32] and workers [33], as well as in patients with CVD [11], McArdle disease [34], severe mental illness [35], and survivors of cancer [36]. 

Our study is the first to extend these findings to show that CRF may mediate the impact of comorbidities on HRQoL in patients with diabetes. The mediating effect of eCRF on the relationship between comorbidities and HRQoL was independent of potential covariates, including demographics (age, sex, marital status), SES (income and education), health behaviors (smoking, alcohol consumption, and regular exercise) and depressive symptoms. In support of the current findings, the findings of the FIT study involving 46,979 patients showed that achieving ≥12 METs reduced the incidence of diabetes compared to achieving <6 METs in both non-obese (hazard ratio = 0.73; 95% CI = 0.59−0.91; *p* < 0.001) and obese individuals (hazard ratio = 0.56; 95% CI 0.42−0.73; *p* < 0.001) [37,38]. In that study [37], the protective effect of achieving high CRF was found to be independent of a number of confounders, including age, sex, race, BMI, history of hypertension, hypertension medication use, ACE inhibitor use, angiotensin II receptor blocker use, b-blocker use, diuretic use, history of hyperlipidemia, lipid lowering medication use, statin use, history of obesity, family history of coronary heart disease, current smoking status, sedentary lifestyle, treated pulmonary disease, depression medication use, and indication for stress testing. Taken together, those findings suggest that high CRF may alleviate the impact of comorbidities on HRQoL in patients with diabetes. Thus, promotion of CRF in conjunction with management of comorbidities targeted at increasing HRQoL should be a key component of the overall management of such patients. 

HRQoL worsens as the number of diabetes-related comorbidities increases [21]. Thus, information regarding determinants and/or mediators of HRQoL is essential for development and implementation of an effective care program to promote physical and mental well-being for older patients with diabetes. Higher levels of physical activity and fitness are associated with lower risks of morbidity and mortality in healthy adults and patients with chronic diseases. Higher CRF is also associated with better HRQoL in healthy adults and in patients with chronic disease. 

The current study has some limitations. First, the cross-sectional study design does not allow causal inference regarding the relationships among comorbidities, eCRF, and HRQoL. A randomized controlled trial on exercise training would be necessary to confirm the mediating effect of CRF on the relationship between comorbidities and HRQoL in a cause-and-effect manner. Second, several potential mediators such as sex, SES, depression, and social networking were identified in previous studies [29]. A more complex mediation model or examination of a larger number of mediation variables should help elucidate the roles of mediator(s) in determining the relationships between exposure and outcomes. Third, the questionnaire of the survey used in this study was not designed to identify the type of diabetes, which should be addressed in a future study. Lastly, a further study will be necessary to investigate the mechanism(s) through which CRF mediates the relationship between comorbidities and HRQoL in diabetes.

## 5. Conclusions

In summary, our findings indicate the mediating effect of eCRF on the relationship between comorbidities and HRQoL in older Korean adults with diabetes. Assessment of eCRF should be considered an important measure for evaluating the overall management of diabetes. From a public health perspective, these findings could help guide public agencies when developing future healthcare policies to promote health equality for geriatric patients with diabetes in South Korea.

## Figures and Tables

**Table 1:** Descriptive statistics of study participants (Mean ± SD).

Variables	All (*n* = 1371)	Men (*n* = 604)	Women (*n* = 767)	*p*-Value
*Demographics*				
Age (years)	69.1 ± 6.1	68.3 ± 5.9	69.8 ± 6.2	<0.001
Marital status, *n* (%)				0.606
Yes	1360 (99.2)	600 (99.3)	760 (99.1)	
No	11 (0.8)	4 (0.7)	7 (0.9)	
Living condition, *n* (%)				<0.001
Live someone	1176 (85.8)	564 (93.4)	612 (79.8)	
Alone	195 (14.2)	40 (6.6)	155 (20.2)	
*Socioeconomic Status*				
Income (10,000 won/month)	228.1 ± 113.0	217.1 ± 319.9	236.8 ± 148.6	0.752
Education, *n* (%)				<0.001
Lower than elementary school	558 (40.7)	122 (20.2)	436 (56.8)	
Middle/high school	597 (43.5)	304 (50.3)	293 (38.2)	
Over than college	216 (15.8)	178 (29.5)	38 (5.0)	
*Health Behavior Factors*				<0.001
Smoking, *n* (%)				
Never	769 (56.1)	87 (14.4)	682 (88.9)	
Past/current	602 (43.9)	517 (85.6)	85 (11.1)	
Weekly alcohol intake, *n* (%)				<0.001
<2	1137 (82.9)	394 (65.2)	743 (96.9)	
≥2	234 (17.1)	210 (34.8)	24 (3.1)	
Regular exercise, *n* (%)				<0.001
Yes	430 (31.4)	243 (40.2)	187 (24.4)	
No	941 (68.6)	361 (59.8)	580 (75.6)	
*Number of Comorbidity, n (%)*				<0.001
0	220 (16.0)	133 (22.0)	87 (11.3)	
1	567 (41.4)	286 (47.4)	281 (36.6)	
≥2	584 (42.6)	185 (30.6)	399 (52.1)	
*eCRF Variables*				
eCRF (METs)	7.1 ± 2.3	9.1 ± 1.6	5.6 ± 1.5	<0.001
BMI (kg/m^2^)	24.7 ± 3.3	24.0 ± 3.0	25.3 ± 3.5	<0.001
RHR (beats/min)	73.0 ± 10.2	72.3 ± 10.5	73.5 ± 9.8	0.023
Physical activity score, *n* (%)				<0.001
Level 1	224 (16.3)	66 (10.9)	158 (20.6)	
Level 2	642 (46.9)	283 (46.9)	359 (46.8)	
Level 3	23 (1.7)	8 (1.3)	15 (2.0)	
Level 4	95 (6.9)	40 (6.6)	55 (7.2)	
Level 5	387 (28.2)	207 (34.3)	180 (23.4)	
*Health-Related Quality of Life*				
EQ-5D problems				
Mobility, *n* (%)	621 (45.3)	189 (31.3)	432 (56.3)	<0.001
Self-care, *n* (%)	205 (15.0)	55 (9.1)	150 (19.6)	<0.001
Usual activities, *n* (%)	409 (29.8)	121 (20.0)	288 (37.5)	<0.001
Pain/discomfort, *n* (%)	585 (42.7)	189 (31.3)	396 (51.6)	<0.001
Anxiety/depression, *n* (%)	260 (19.0)	79 (13.1)	181 (23.6)	<0.001
EQ-5D index	0.85 ± 0.18	0.90 ± 0.15	0.81 ± 0.19	<0.001
EQ-VAS score	67.0 ± 22.1	70.1 ± 19.0	64.6 ± 24.0	<0.001

SD—standard deviation; eCRF—estimated cardiorespiratory fitness; MET—metabolic equivalents; BMI—body mass index; RHR—resting heart rate; EQ-5D—euroqol-5 dimension; EQ-VAS—euroqol-visual analogue scale.

**Table 2:** Comparison of measurement variables according to number of comorbidity (Mean ± SD).

Variables	Number of Comorbidity			*p* for Linear Trend
	0 (*n* = 220)	1 (*n* = 567)	≥2 (*n* = 584)	
*Socio-Demographic Status*				
Age (years)	67.6 ± 6.0	69.3 ± 6.3	69.6 ± 5.9	<0.001
Marital status, *n* (%)				0.194
Yes	219 (99.5)	564 (99.5)	577 (98.8)	
No	1 (0.5)	3 (0.5)	7 (1.2)	
Living condition, *n* (%)				0.022
Live someone	197 (89.5)	491 (86.6)	488 (83.6)	
Alone	23 (10.5)	76 (13.4)	96 (16.4)	
*Socio-Economic Status*				
Income (10,000 won/month)	236.5 ± 386.3	268.0 ± 172.2	186.8 ± 289.2	0.583
Education, *n* (%)				0.001
Lower than elementary school	71 (32.3)	234 (41.3)	253 (43.3)	
Middle/high school	106 (48.2)	234 (41.3)	257 (44.0)	
Over than college	43 (19.5)	99 (17.4)	74 (12.7)	
*Health Behavior Factor*				
Smoking, *n* (%)				<0.001
Never	102 (46.4)	285 (50.3)	382 (65.4)	
Past/current	118 (53.6)	282 (49.7)	202 (34.6)	
Weekly alcohol intake, *n* (%)				0.004
<2	181 (82.3)	444 (78.3)	512 (87.7)	
≥2	39 (17.7)	123 (21.7)	72 (12.3)	
Regular exercise, *n* (%)				0.251
Yes	79 (35.9)	172 (30.3)	179 (30.7)	
No	141 (64.1)	395 (69.7)	405 (69.3)	
*HRQoL*				
EQ-5D problems				
Mobility, *n* (%)	69 (31.4)	221 (39.0)	331 (56.7)	<0.001
Self-care, *n* (%)	24 (10.9)	58 (10.2)	123 (21.1)	<0.001
Usual activities, *n* (%)	41 (18.6)	136 (24.0)	232 (39.7)	<0.001
Pain/discomfort, *n* (%)	73 (33.2)	209 (36.9)	303 (51.9)	<0.001
Anxiety/depression, *n* (%)	33 (15.0)	93 (16.4)	134 (22.9)	<0.001
EQ-5D index	0.90 ± 0.14	0.87 ± 0.16	0.81 ± 0.20	<0.001
EQ-VAS score	69.2 ± 22.7	69.4 ± 20.9	63.9 ± 22.5	0.002

**Table 3:** Descriptive statistics of measured parameters according to eCRF categories (Mean ± SD).

Variables	eCRF Categories			*p* for Linear Trend
	Low (*n* = 342)	Middle (*n* = 686)	High (*n* = 343)	
eCRF (METs)	5.3 ± 1.8	7.0 ± 1.9	9.3 ± 1.8	<0.001
*Demographics*				
Age (years)	74.3 ± 5.5	67.9 ± 5.3	66.5 ± 5.2	<0.001
Marital status, *n* (%)				0.198
Yes	339 (99.1)	678 (98.8)	343 (100.0)	
No	3 (0.9)	8 (1.2)	0 (0.0)	
Living condition, *n* (%)				0.003
Live someone	274 (80.1)	600 (87.5)	302 (88.0)	
Alone	68 (19.9)	86 (12.5)	41 (12.0)	
*Socioeconomic Status*				
Income (10,000 won/month)	291.4 ± 220.3	191.7 ± 253.6	238.0 ± 418.4	0.547
Education, *n* (%)				0.002
Lower than elementary school	155 (45.3)	288 (42.0)	115 (33.5)	
Middle/high school	144 (42.1)	284 (41.4)	169 (49.3)	
Over than college	43 (12.6)	114 (16.6)	59 (17.2)	
*Health Behavior Factors*				
Smoking, *n* (%)				0.676
Never	186 (54.4)	391 (57.0)	192 (56.0)	
Past/current	156 (45.6)	295 (43.0)	151 (44.0)	
Weekly alcohol intake, *n* (%)				0.034
<2	301 (88.0)	555 (80.9)	1.9)	
≥2	41 (12.0)	131 (19.1)	62 (18.1)	
Regular exercise, *n* (%)				<0.001
Yes	83 (24.3)	216 (31.5)	131 (38.2)	
No	259 (75.7)	470 (68.5)	212 (61.8)	
*Number of Comorbidity, n (%)*				0.008
0	36 (10.5)	115 (16.8)	69 (20.1)	
1	156 (45.6)	270 (39.4)	1.1)	
≥2	150 (43.9)	301 (43.8)	133 (38.8)	
*Health-Related Quality of Life*				
EQ-5D problems				
Mobility, *n* (%)	206 (60.2)	288 (42.0)	(37.0) 127	<0.001
Self-care, *n* (%)	85 (24.9)	85 (12.4)	35 (10.2)	<0.001
Usual activities, *n* (%)	141 (41.2)	178 (25.9)	90 (26.2)	<0.001
Pain/discomfort, *n* (%)	170 (49.7)	271 (39.5)	144 (42.0)	0.041
Anxiety/depression, *n* (%)	64 (18.7)	140 (20.4)	56 (16.3)	0.425
EQ-5D index	0.80 ± 0.22	0.87 ± 0.16	0.87 ± 0.16	<0.001
EQ-VAS score	62.9 ± 24.9	67.3 ± 20.9	70.6 ± 20.7	<0.001

**Table 4:** The association between comorbidity and health related quality of life, mediated by eCRF, in diabetes.

Path	Model 1		Model 2		Model 3	
	β (SE)	95% CI	β (SE)	95% CI	β (SE)	95% CI
Comorbidity → eCRF, a	−0.528 (0.063) **	−0.652 to −0.404	−0.388 (0.055) **	−0.495 to −0.281	−0.244 (0.046) **	−0.335 to −0.154
eCRF → Quality of life, b	1.434 (0.258) **	0.928 to 1.940	0.996 (0.300) **	0.407 to 1.586	1.153 (0.358) *	0.452 to 1.855
Total effect, c	−2.670 (0.611) **	−3.868 to −1.472	−2.327 (0.609) **	−3.522 to −1.133	−2.321 (0.613) **	−3.522 to −1.119
Direct effect, c′	−1.913 (0.619) *	−3.128 to −0.698	−1.941 (0.618) *	−3.153 to −0.729	−2.039 (0.617) *	−3.249 to −0.829
Indirect effect, ab	−0.757 (0.168)	−1.104 to −0.453	−0.387 (0.135)	−0.672 to −0.145	−0.282 (0.107)	−0.517 to −0.095
Ratio of indirect to total effect mediated (%)	28.4		16.1		12.1	
Sobel test	−4.632 **		−3.004 **		−2.753 **	

Model 1 was non-adjusted. Model 2 was adjusted for age, marital status, living condition, and education. Model 3 was adjusted for Model 2 + smoking, weekly alcohol consumption, and regular exercise. Number of bootstrap samples for bias-corrected bootstrap confidence intervals: 5000. eCRF—non-exercise-based estimation of cardiorespiratory fitness; CI—confidence interval; SE—standard error. * *p* < 0.005, ** *p* < 0.001.
